# A decentralised architecture for secure exchange of assets in data spaces: The case of SEDIMARK

**DOI:** 10.1016/j.dib.2025.111757

**Published:** 2025-06-10

**Authors:** Erika Duriakova, Diarmuid O’Reilly-Morgan, Maroua Bahri, Maxime Costalonga, Gabriel Danciu, Septimiu Nechifor, Stefan-Cristian Jarcau, Tarek Elsaleh, Peipei Wu, Franck Le Gall, Grigorios Koutantos, Panagiotis Vlacheas, Luis Sánchez, Juan Ramón Santana, Pablo Sotres, Elias Tragos

**Affiliations:** a Insight Research Ireland Centre for Data Analytics, Beech Hill, University College Dublin, Belfield, Dublin 4, Ireland; b Sorbonne Université, CNRS, LIP6, Inria, 75005, Paris, France; c Network Planning and Mobile Communications Lab, Universidad de Cantabria, Edificio Ingeniería de Telecomunicación, 39005, Santander, Spain; d Centre for Vision, Speech and Signal Processing, University of Surrey, Guildford GU2 7XH, United Kingdom; e EGM, 1 Traverse des Brucs, 0560 Valbonne, France; f Eviden BDS R&D Spain, Calle Albarracín 25, Madrid 28037, Spain; g Siemens Romania R&D, Bulevardul 15 Noiembrie 78 AFI Park 1, Brașov, Romania; h WINGS ICT Solutions S.A, 189, Siggrou Avenue, 17121, Athens, Greece

**Keywords:** Decentralised data spaces, System architecture, Marketplace, Data quality, Artificial intelligence

## Abstract

The European Union's (EU) data strategy aims to create a single market for seamless data flow while ensuring proper governance, privacy, and data protection. In this paper, we present SEDIMARK, an EU project, that builds on this strategy by developing a fully decentralised, secure data marketplace.

The goal of SEDIMARK is to build a complete toolbox that enables users to purchase and process data assets. The toolbox includes tools for data cleaning, decentralised machine learning models and secure data exchange. SEDIMARK offers users full control over data assets by enabling them to keep their data locally and thus removing the need for central servers. With customisable pipelines and tools, SEDIMARK supports a wide range of users, from novices to experts, promoting seamless collaboration and fair access to high-quality datasets across Europe.

The decentralised connectivity in SEDIMARK is achieved with the use of Distributed Ledger Technology (DLT). Furthermore, SEDIMARK’s architecture features a unique Connector component using Self Sovereign Identities (SSI), fostering trust and secure interactions. Transactions in SEDIMARK are stored in a Registry, a decentralised, immutable, non-repudiable and permissionless database. Together the technologies used in SEDIMARK ensure privacy, trust and data quality for secure management, sharing, and monetisation of assets in data spaces.

## 1 Background

Data has become a new currency in the last decade. It is crucial for companies and researchers to have access to high-quality diverse datasets to promote research and develop more advanced and user-tailored products and services. Even though many attempts have been made to create data repositories, especially within the EU, they are mostly centralised, which might discourage data providers that have confidential data to easily share them with their peers. Additionally, central repositories rarely provide functionalities for improving or assessing data quality and usually rely on users’ self-assessment of their data. The motivation for this work was to provide an architectural framework for a completely decentralised data marketplace, building on the EU’s concept of data spaces, while leveraging Distributed Ledger Technology for increased trust. This architecture aims to provide users with functionalities for sharing their data (while keeping them locally on their premises), improving and assessing the data quality, building AI models either locally or in a distributed way using distributed machine learning and providing interoperability for re-using datasets.

## 2 Introduction

The EU has recently laid out a “data strategy” [1], aiming to position itself as a leader in the transition towards data-driven societies. The EU vision is to create a “single market” that enables the free flow of data within the EU, helping researchers, businesses and administrations to create better services and products. However, this free flow of data should be properly governed and abide by the EU rules and regulations for FAIR access, re-usability and above all privacy and data protection. In this respect, the EU has also set out specific rules through the EU Data Act [2], with new measures to make more data available for the benefit of EU organisations.

A pillar of the EU data strategy is the creation of interoperable EU data spaces in various sectors to overcome the issues arising around data sharing. Subsequently, the development of infrastructure and policies for the exchange of data between organisations requires significant investments in intelligent tools for interoperability, secure data sharing, data discovery and data processing. Many initiatives have been established to help develop architectures for data exchanges, i.e. Big Data Value Association (BDVA) [3], and International Data Spaces Association (IDSA) [3], focusing more on the establishment of policies and procedures among participants to allow the secure exchange of data between them. Lately, the EU has also set up the Data Spaces Support Centre (DSSC) [4] to provide the foundations for common requirements, practices and architectures for the development of EU data spaces.

Acknowledging that the EU data economy is growing rapidly and is estimated to reach 800 billion Euros by 2025 [5], the architectures for data spaces should not only focus on policies and procedures for data exchange but also on techniques to allow extracting more value from the data available within and across the data spaces. Thus, efficient data management techniques should be developed to boost the accessibility and discoverability of high-quality, secure and privacy-enhanced data, especially in the context of EU Data Spaces. Considering also the need for enhanced data privacy and user data control, decentralisation should be promoted, allowing advanced distributed data management techniques at the “edge”.

SEDIMARK [6] is an EU project that develops an architecture for a secure and decentralised data and services marketplace. SEDIMARK’s vision will facilitate the generation, curation, discovery and enrichment of scattered data from various domains so that their value increases significantly before they are either exchanged in the marketplace or exploited in the form of Machine Learning (ML) services. The added benefit of SEDIMARK is that it is a fully decentralised platform built on top of the IOTA DLT [7] and focused on enhancing the value of data before they become assets to be exchanged. In this paper, we provide the main concepts of SEDIMARK, its functional architecture, and how researchers and companies can use the SEDIMARK platform to share data of high value and quality. In summary, our novel contributions include the following:

• Developed an architecture capable to facilitate data pooling and sharing within the European strategy for data specifically focusing on decentralised data sharing solution that goes beyond decentralised data storage
• At the core of SEDIMARK’s architecture, we provide advanced tools for decentralised approaches in data curation, data quality enhancement and AI-based data processing
• Our architecture design offers true decentralised and trustworthy solution for data assets and AI-based assets publication and exchange leveraging Distributed Ledger Technology for increased trust.
• Through detailed analysis and initial evaluation, we demonstrate the potential of our architecture to fill in the gap of a fully decentralised data marketplace.

The rest of this article is organised as follows: Section 2 describes related work in data space research advancements and shows how SEDIMARK improves upon state-of-the-art approaches; Section 3 introduces the high-level view of the SEDIMARK’s architecture; Section 4 describes individual architectural components in greater detail and demonstrates how the components fit together; and finally, we close this article in Section 5.

## 3 Related Work

The goal of recent advances in data space research is to address fair access to data and support decentralisation. This section provides an overview of the state of play of the Common European Data Spaces and the main findings that have emerged from these initiatives. Furthermore, the technical details of the latest data space architectures together with data marketplaces and their relevance to our work.

### 3.1 Common European data spaces

The European Union's Digital Europe Programme (DIGITAL) has initiated several projects to develop sector-specific data spaces (e.g. Agriculture, Cultural Heritage, Energy, Green deal, Health, Tourism, etc.) aiming to enhance data sharing and interoperability across various domains. Notable findings and developments from these projects are being fed into the evolution of aforementioned initiatives, which are leading the technical development and evolution. In this sense, while the specific needs of each sector may vary, there are several common findings emerging from the different data space initiatives across various sectors (e.g., agriculture, mobility, cultural heritage). These common insights reveal trends and challenges that are consistent across the diverse projects being rolled out. These common findings reflect the shared challenges and priorities of data space projects across Europe.

Key themes include interoperability, trust, scalability, sustainability, and a focus on legal and ethical frameworks. Firstly, there is a strong emphasis on the need for interoperability between different systems, platforms, and data sources. Ensuring that data can be seamlessly shared across diverse platforms requires standardized frameworks, protocols, and formats.

Secondly, data sovereignty remains a key concern across all data space initiatives. Stakeholders want to retain control over their data and make decisions about how it is shared. This is particularly critical in sectors where sensitive data is involved. Besides the technical aspects, many projects highlight the need for clear legal frameworks to manage data sharing, ensuring compliance with the GDPR and other privacy regulations. This includes addressing data protection issues and establishing protocols for data access and usage that comply with national and international laws.

An aspect that is particularly gaining attraction is the potential of Artificial Intelligence (AI) as a key enabler for unlocking the potential of data spaces. Many of the projects involve using AI to process, analyse, and enrich data to make it more useful for stakeholders. In this regard, an aspect that has been typically neglected relates with data quality and metadata management. High-quality, well-structured data is essential for making data spaces effective.

Furthermore, Data Spaces are promoted to avoid the siloed situation that has predominated till the moment. Thus, the creation of larger silos should be avoided. Hence, successful data spaces are not limited to one sector; they require collaboration between different stakeholders, including businesses, governments, research institutions, and civil society. Building a robust ecosystem around these data spaces—comprising diverse participants—has been identified as a crucial success factor. This collaboration helps to promote innovation and scale data-driven solutions. Eventually, a strong focus is being put on ensuring that data spaces that are being rolled-out can scale across different regions, countries, and sectors. Sustainability, both in terms of funding and long-term operational viability, is a key consideration.

Finally, ensuring that data spaces are accessible to a broad range of users, including small businesses and non-experts, is another critical finding. The user interface and experience need to be designed with accessibility in mind, ensuring that stakeholders across industries can leverage the data efficiently and intuitively. In this regard, a common technical challenge across various initiatives is the creation of robust infrastructure, including secure data exchange platforms and middleware solutions. For example, the SIMPL^1^ framework, developed for cross-sector data spaces, aims to support interoperability, secure data sharing, and seamless integration across European data spaces.

SEDIMARK is actually addressing some of the aspects and challenges that emanate from the practical work of sectoral Data Spaces that are being rolled out. Fundamentally, through the development of data and metadata models as well as leveraging common protocols and standards SEDIMARK is tackling interoperability aspects. Moreover, by rooting SEDIMARK’s architecture on de-centralized and distributed principles, it is promoting data sovereignty and cross-sectorial ecosystem creation. Indeed, the system that is described in this paper is being deployed to support four use-cases on three different sectors (mobility, energy and water). Furthermore, SEDIMARK’s system addresses the importance of data quality and metadata management embedded within the application and support of advanced AI techniques such as Federated Learning and is developing solutions on these aspects.

### 3.2 Data spaces architectures

The current technology landscape of data spaces is evolving at a dazzling pace. Current efforts aim to provide a harmonised set of rules to ensure fair access to data. The most mature solution in terms of existing technical developments relates to the International Data Space (IDS) proposal by the International Data Spaces Association (IDSA). The second generation of data space technologies, like Gaia-X and the Blueprint specified by the Data Spaces Support Centre (DSSC) [4] are further fostering the decentralised nature of data spaces by reducing dependency on central components.

The IDS architecture provides a standardised reference framework for decentralised data exchange, including common domain-agnostic data models and protocols [8]. The architecture is centred around the notion of a *Connector*, supported by a set of centralised components provided by a data space facilitator. Connectors carry out all interactions in a data space and establish secure and trustworthy communications between participants. Before data exchange can take place, participants’ Connectors must sign a legally binding contract establishing the conditions under which data can be accessed and used. At present, IDSA is engaged in the development of a new standard, the Dataspace Protocol [9], which represents a more flexible approach, defining control mechanisms for the data exchange, but not for data transfer itself.

Gaia-X specifies a framework for creating a federated and secure data infrastructure based on a common trust framework [10], where all stakeholders and product offerings have self-descriptions supported by verifiable credentials, which are issued by trust anchors endorsed by Gaia-X. In a Gaia-X data space, all data is kept by the data provider, and it is only exchanged after negotiating an agreement with the data consumer that specifies how the data can be used. For this purpose, Self-Sovereign Identities (SSI) are the basis to support digital identities. Gaia-X defines a set of federation services with a focus on compliance services, on which the trust framework is built. These services include the functionality needed for describing, managing, and discovering data exchange services, as well as negotiating the exchange of data.

DSSC is a recently funded project aiming to set up and operate a support centre for data spaces, aligned with the European Strategy for Data. Hence, the DSSC aims to establish shared data spaces that collectively foster an interoperable environment for data sharing, allowing the reuse of data in several sectors. The DSSC works on defining its own Conceptual Model, aiming to organise the fundamental concepts and terms associated with data spaces, establishing a standardised vocabulary that ensures clear communication and shared understanding among stakeholders, to enable consistent interpretations and reduce the ambiguity involved in discussing data spaces across different contexts. The DSSC promotes a definition for a data space, which states that: “A Data Space is an infrastructure that enables data transactions between different data ecosystem parties based on the governance framework of that data space” [11]. To realise that, the DSSC has defined its *Blueprint*, consisting of several *building blocks* to be considered when constructing a data space. These building blocks are arranged around three main pillars: Interoperability, Trust and Data value, and delineate key areas where choices are required to enable effective and trusted sharing of data among participants.

The SIMPL framework is a critical infrastructure being developed to support the interoperability and secure data sharing within the European data spaces. It is part of the European Commission's efforts under the EU Digital Strategy to enable seamless and trusted data exchange across different sectors and regions. SIMPL aims to create a common infrastructure that facilitates the secure, interoperable, and scalable exchange of data among the various data spaces across Europe.

SIMPL focuses on supporting interoperability, security, privacy and data sovereignty, including mechanisms to ensure proper governance and legal compliance for data sharing. It leverages a middleware platform, acting as a bridge between different data systems. It is being designed to be scalable, enabling it to handle the growing volume of data across various sectors. It can support both small-scale and large-scale data exchanges, making it suitable for different types of organizations, from SMEs to large enterprises and governmental bodies. SIMPL is not meant to be just a technological solution but is part of a broader ecosystem aimed at supporting the development of data-driven services and innovation across Europe.

As it has been mentioned SEDIMARK is rolling out its marketplace ecosystem, which stands on the Data Spaces baseline, to support actual use-cases. In this sense, since the project has been following existing parallel initiatives, the outcomes from the project could be further integrated into the common frameworks that are being created in DSSC and SIMPL.

### 3.3 Marketplaces

In recent years a number of projects have proposed frameworks for decentralised marketplaces. In particular, we can highlight the following projects as described in the next few paragraphs.

The i3-MARKET project^2^ is a comprehensive data management initiative to create a secure and robust single European data market. It uses a federation of data marketplaces to allow secure and privacy-preserving data sharing across multiple platforms. This project primarily focuses on industrial data, addressing the lack of trusted and secure solutions for the federation of data marketplaces. The data storage system in i3-MARKET has a two-fold architecture, incorporating both decentralised and distributed storage. These storage systems handle data like identity information, shared semantic models, meta-information about datasets, semantic queries, and smart contract templates. They are implemented through technologies such as Hyperledger Besu^3^ and CockroachDB.^4^

KRAKEN [12] is an EU project that focuses on building a personal data platform for sharing potentially sensitive personal data. The architecture of KRAKEN is decentralised, based on the concepts of SSI and cryptography, including also a marketplace for the exchange of sensitive data between providers and consumers.

However, these two projects are not aligned with the European Data Spaces ecosystem that is being pushed to facilitate data pooling and sharing within the European strategy for data. Moreover, both i3-MARKET and KRAKEN fail to provide true decentralised and trustworthy solutions for data assets and AI-based assets publication and exchange, as they rely on partly centralised catalogues and registries. Besides, they also fail to provide advanced tools for data curation, data quality enhancement and AI-based data processing which are at the core of SEDIMARK’s platform value proposition, thus integrated within each SEDIMARK Marketplace participant’s Toolbox.

Finally, The OMEGA-X marketplace [13] is built upon concepts originating from IDSA and Gaia-X and aims to build a complete Data Space focused on the energy sector. The OMEGA-X architecture includes a component called “The Data & app marketplace”, which handles users' identities, registration and login, as well as the negotiation of contracts between providers and consumers. OMEGA-X also employs the concept of a connector, enabling data exchanges and service provision using a Federated Catalogue. However, OMEGA-X identity management and contract negotiation are still based on centralised solutions. On the contrary, SEDIMARK’s platform enables complete decentralisation, not only in terms of storage of the assets to be exchanged but also in terms of the trust framework that has to be created among all the participants of the marketplace. This way the whole asset lifecycle (from publication to negotiation and exchange) happens in a distributed manner with trustworthiness rooted in the use of SSI and smart contracts available at a DLT network.

## 4 SEDIMARK Architecture

The overall SEDIMARK’s architecture consists of 2 main building blocks as follows: (i) SEDIMARK requirements designed to guide the overall process of building the SEDIMARK’s architecture; and (ii) the functional architecture itself. In the next few paragraphs, we describe both building blocks in more detail.

### 4.1 SEDIMARK requirements

SEDIMARK aims to build a data and services marketplace based on specific identified functional and non-functional requirements that guide the development process of the various modules [14]. SEDIMARK’s main non-functional requirements are for the architecture to be decentralised, scalable and provide features for security, privacy and trust, so that the users of the system feel “safe” when sharing their data or purchasing datasets. Additionally, the architecture should support intelligence (via functionalities for training and running machine learning and artificial intelligence models both locally and in a decentralised way) and energy efficiency to decrease the carbon footprint of data management, processing and sharing mechanisms. Additionally, interoperability and extensibility are also very important requirements for SEDIMARK so that data is shared in interoperable formats, making it easy to reuse, allowing the easy extension of the system with new modules to support more functionalities in the future. The functional requirements of SEDIMARK are mainly focusing on setting the needs for the functional modules of the system, dealing with i.e. data cleaning and curation tools, the ability to support both streaming and static data, providing tools for building intelligence out of shared data, trusted data sharing, decentralised and secure data storage, global and local catalogues of assets, required offering metadata (so that users can easily discover assets), asset tokenization, user digital wallets, etc. All these features are equally important to allow the users to extract maximum value from the data while maintaining their privacy and full user control.

### 4.2 Functional architecture

**Fig. 1:**
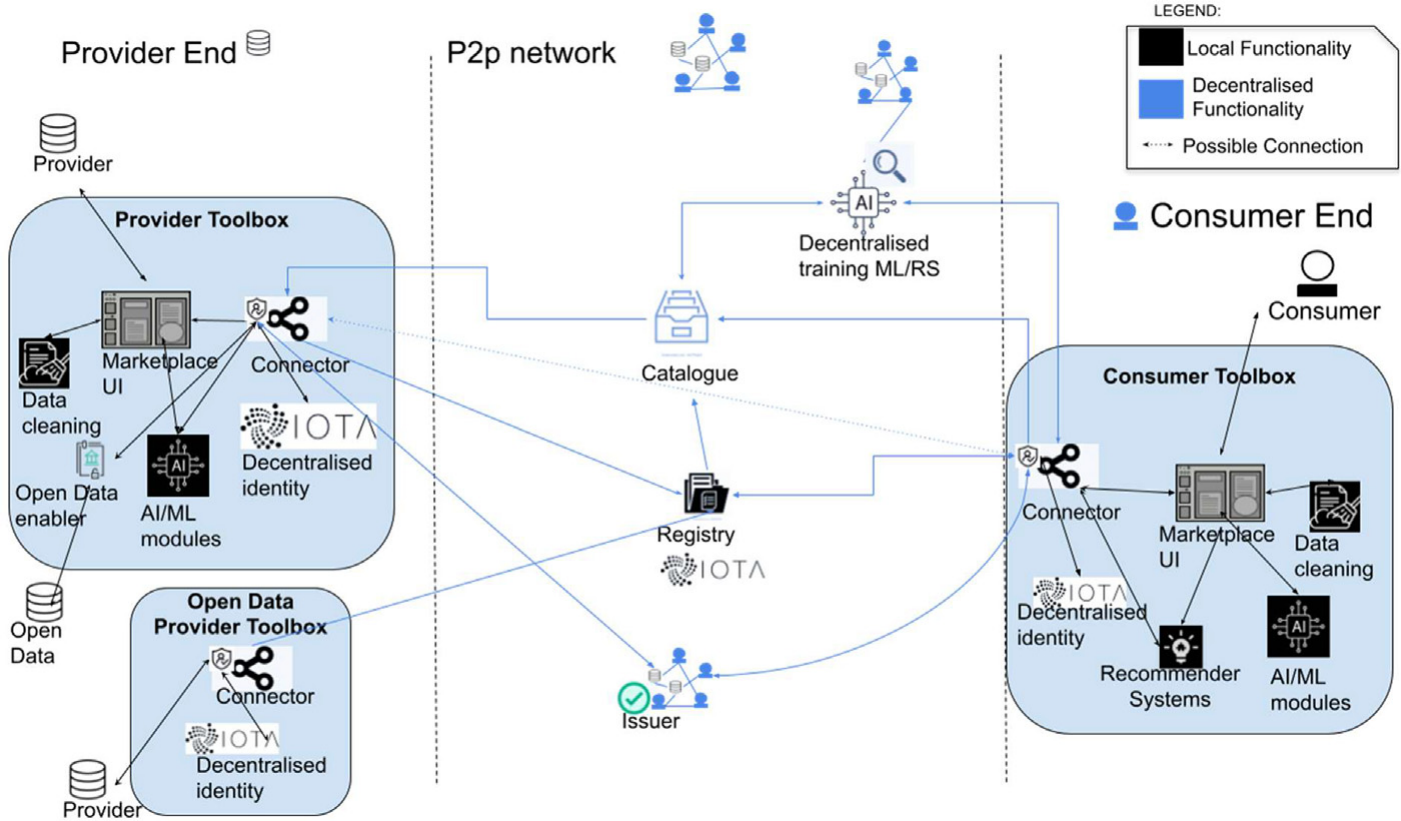
SEDIMARK high-level architecture [15].

SEDIMARK’s vision is to provide a complete toolbox for enabling researchers, companies, organisations or individuals to purchase and process data, assess and improve their quality, build machine learning models and share data and services with other organisations across Europe. SEDIMARK removes the need for any central servers to store data/assets or maintain lists of shared assets and promotes the full decentralisation of data spaces so that users can have full control over their assets and manage who, how and for how long they will get access to these assets. The main high-level conceptual architecture of SEDIMARK is shown in Fig. 1 [15]. The decentralisation of the architecture is evident through the lack of any centralised component, while it relies on a peer-to-peer network in the form of a DLT.

Any user of the SEDIMARK marketplace is called a *Participant* and what they exchange and share through the marketplace are called *assets* (see also Section 4.4). As can be seen in the figure, *Participants* are split into two main roles: (i) *Providers* and (ii) *Consumers. Providers* are users who engage in sharing their assets through the marketplace and will get access to all of the necessary tools for managing their access and converting them into offerings to be shared through the marketplace. *Consumers* are users that browse the marketplace, discover and request access to *assets* that are of interest to them, i.e. request datasets in various formats or access services and analytics. It has to be noted that a SEDIMARK *participant* can also have a dual role, being both *Provider* and *Consumer* at the same time.

Any *Participant* will be able to download the SEDIMARK toolbox, which will be dynamically built based upon the role of the *Participant* and their intended toolbox usage. The dynamic nature of the toolbox will further allow *Participants* to add extra modules as required, expanding the potential services that they can access. For example, *Providers* will download a version of the toolbox that will include the modules for data processing and curation, and for creating offerings and sharing them through the marketplace. On the other hand, the Consumer toolbox will include modules to browse the marketplace, discover assets, get recommendations for assets, and purchase them. Both roles can further expand their toolboxes with modules for training and processing AI models, either locally or distributedly.

A central component of the SEDIMARK architecture is the “*Connector*”, which contains all the functionalities necessary to connect users through the IOTA DLT. For this connectivity, SEDIMARK uses a component to create decentralised identities based on the concept of Self Sovereign Identities (SSI) and Decentralised Identifiers (DIDs), giving users full control over the information they use to prove who they are. *Providers* have access to a Data Curation Pipeline, which includes AI-based components for assessing and improving data quality, including tools for dataset deduplication, outlier detection and removal, data augmentation, missing value imputation, etc. Using these tools, *Providers* will be able to optimise their data and share high-quality datasets in the marketplace, increasing their value and potential for exploitation. The data pipeline also includes components for formatting the data into suitable formats for processing and interoperability, converting the datasets to common interoperable formats, i.e. NGSI-LD [16], to maximise the potential for reuse in various ways.

The *Consumer* toolbox includes the User Interface and the respective components to browse the marketplace, discover assets based on their queries, and receive personalised recommendations for assets based on user history and preferences. To maintain its appeal, the SEDIMARK catalogue will be continuously populated by free offerings, accessible to all participants, granting them access to open datasets or APIs directly in the marketplace. Both versions of the toolbox may also include the AI pipeline, which is a set of tools that allow the training of AI models based on assets, which users either generated locally or purchased from the marketplace. SEDIMARK improves upon most data space architectures by providing components for training AI models in a decentralised way, using techniques such as Federated Learning, Gossip Learning, or Split Learning. SEDIMARK pipelines are fully customisable and can be tailored to the preferences and the technical expertise of users, allowing even novice users to deploy template pipelines, while expert users will be able to customise the settings of the modules, i.e. change values of parameters, which models to be used, etc.

Any transaction that takes place within SEDIMARK is stored in the *Registry*, which takes the form of a decentralised, immutable, non-repudiable and permissionless database. Providers register their assets in the registry, providing descriptions of their offerings so that they will be discoverable by consumers. Each Provider has a local listing of their assets, which can be federated with the listings of other providers, helping form the “Catalogue”, which is a semantically searchable distributed database constructed by crawling the registry to find new offerings and then getting the information from the local listings of the providers.

One important component of the SEDIMARK architecture is the Open Data enabler, which allows the connectivity of the SEDIMARK platform with external open data repositories so that Consumers can also search and get access to external non-SEDIMARK assets. The following sections of this paper provide more details about the functional components of SEDIMARK.

The goal of SEDIMARK is to allow the providers to share their data while still keeping them locally and having full control over who has access to them. The fact that providers will keep their data locally enables them to keep using their own existing infrastructures where they store and process the data they collect. The SEDIMARK toolbox, through the data curation pipeline and the SEDIMARK connector ensures the smooth integration of the provider's existing infrastructure with the SEDIMARK marketplace. The data curation pipeline includes data loaders and interoperability components that will connect the SEDIMARK platform with the providers' infrastructures, so that their data are injected into the SEDIMARK data processing pipeline to be converted to interoperable formats before being shared in the marketplace. Several drawbacks can arise on the side of providers including the following: (i) providers will need to download and install the SEDIMARK toolbox in order to share their data on the marketplace; (ii) if the data format of the data provider is not supported by SEDIMARK marketplace, the provider will need to implement a data mapper that can convert the data to one of the data formats recognised by SEDIMARK; (iii) data providers will need to account for additional storage space, since the data pipeline converts the data to NGSI-LD format (for interoperability).

## 5 SEDIMARK Technologies

As specified in earlier sections, SEDIMARK leverages the concept of EU data spaces with a focus on decentralising data assets while ensuring trust, intelligence, interoperability, and data quality. Therefore, the SEDIMARK architecture comprises multiple components designed to address specific aspects of these goals, facilitating secure and efficient data exchange within a decentralised marketplace. In the following sections, we describe each component in greater detail.

### 5.1 Decentralisation supported by DLT

Following the principles of decentralisation, SEDIMARK addresses the growing demand for secure and transparent data exchange in the global digital economy. Using DLT, the SEDIMARK platform establishes a resilient and scalable infrastructure able to provide trustworthy, non-repudiable and immutable information about participants and offerings within the marketplace. In this sense, the decentralised architecture features a robust distributed ledger for managing user identities and a blockchain foundation to foster tamper-resistant contracts. The use of a distributed network eliminates a single point of failure, thereby enhancing the overall reliability of the system and ensuring the continuous availability of marketplace assets. Moreover, the decentralised infrastructure supports standardised protocols for data exchange, enabling seamless collaboration and data sharing across multiple platforms and participants.

From an architectural point of view, the SEDIMARK decentralised marketplace leverages the IOTA DLT infrastructure, which can be divided into two interacting layers. Layer 1 (L1) comprises the IOTA ledger, also known as the Tangle [7], while Layer 2 (L2) contains the IOTA Smart Contracts framework [17]. L1 is used for managing participant identities as well as for anchoring L2 smart contract chains, while L2 handles the core business logic of the SEDIMARK marketplace, enabling trustworthy offering discovery and trading. Information within these layers is distributed over a meshed network of nodes to allow decentralised access. In addition, participant identities follow the self-sovereign identity paradigm using decentralised identifiers (DIDs) [18] and Verifiable Credentials (VCs) [19].

**Fig. 2:**
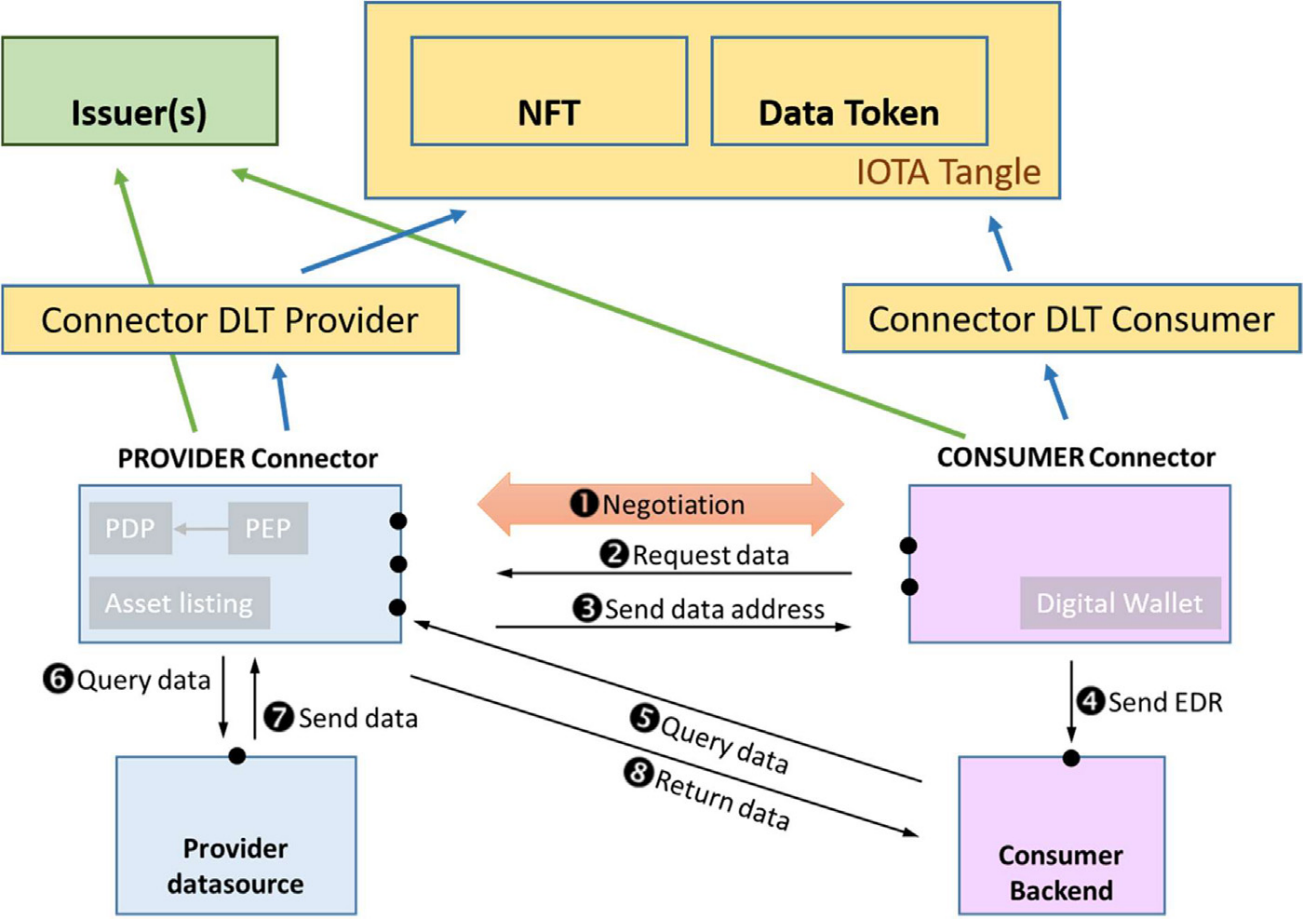
DLT-based asset exchange architecture.

Fig. 2 depicts the interconnection of the DLT-related infrastructure, represented by the *Tangle* and the *Issuer(s)*, and the gateway components within each participant domain, known as *Connectors*. The figure shows the result of the tokenization of an offering in L2, which is represented by a single Non-Fungible Token (NFT) and a set of Data Tokens (DTs). While the NFT provides trustworthy and immutable information that can later be used to build offering catalogues, DTs, which are linked to the NFT, are exchanged between the corresponding provider and consumers during the offering negotiation process. In addition, connectors rely on the Issuer(s) for credential validation.

The decision to use NFTs and the associated DTs is mainly motivated by the fact that, this way, both the process of creation of an offering by a Provider and the process of purchase of the asset associated with such an offering by a Consumer, can be managed through smart contracts that are deployed at the DLT network. In this sense, both processes are ruled by respective smart contracts implemented at L2 of the IOTA Tangle. Besides automatising and streamlining such key marketplace processes, the decentralisation and auditability of such combined infrastructure (i.e. DLT and smart contracts) provides a solid ground for guaranteeing the trustworthiness among the marketplace participants that these participants demand. Moreover, the immutability associated with the subjacent technology employed by the SEDIMARK marketplace guarantees that liability can always be enforced in case of occurrence of a dispute among the parties involved in any asset transaction.

As a result, and from a functional perspective, this infrastructure serves as a foundation to support the following functionalities: i) management of participant identities; ii) offering metadata management for catalogue generation; iii) storage of trust metadata; iv) tokenisation of offerings; v) secure trading of assets among participants; and vi) interactions with participants’ wallets.

### 5.2 AI-based tools for data processing and interoperability

One of the key components of the SEDIMARK architecture is the Data Processing Pipeline (DPP), which aims to support the management of the “quality” of data assets. The DPP is a group of components that provide support to users to understand their data, assess their quality, and curate them, increasing both the data's quality and its value before they are either shared in the marketplace or further processed within the AI pipeline.

The SEDIMARK DPP has been designed based on the concepts of modularity and extensibility. The DPP is a modular pipeline, consisting of many components which can either work independently serving specific goals, or can be grouped for use in more complex data processing scenarios. This flexible plug-and-play pipeline allows for personalised data cleaning with user-defined specific data cleaning steps (and their order). The pipeline is specifically designed so that all the modules use the same input and output formats, and so can function together in any (reasonable) order. For example, a user may decide to first run data deduplication, to remove duplicate values, and then run outlier detection, while another user might want to run the modules in the opposite order.

The SEDIMARK DPP is built in Python, with interfaces supporting the extension of the pipeline with new modules or new ML models. For example, the outlier detection module is agnostic to the model used for detection, and a user can add a new model as long as the model is invoked by the module in the same way as the base models supplied by SEDIMARK's DPP, similarly as in the PyOD [20] or TODS [21] libraries. Additionally, SEDIMARK DPP aims to cater to both novice users who want to use default pipelines with pre-set values, and expert users who can configure every step and module in the pipeline.

For facilitating the minimum possible human intervention and the automation of the overall pipeline, SEDIMARK also works on implementing state-of-the-art Automated Machine Learning (AutoML) techniques. AutoML will be used for selecting the most appropriate models used in the pipeline, choosing the best hyperparameter values for those models, and for feature engineering, etc. [22]. Using libraries such as auto-sklearn and TPOT [23], the platform automatically selects the most appropriate machine learning model (and their hyperparameter values) for the data processing steps based on the characteristics of the dataset and user preferences. Existing AutoML tools are predominantly designed for static datasets and are unable to handle data streams [24]. Innovative solutions will be developed to address this limitation in the context of SEDIMARK's data streams. These will extend current methodologies for data cleaning and also real-time AI-driven analytics, ensuring continuous and efficient processing of streaming data.

**Fig. 3:**
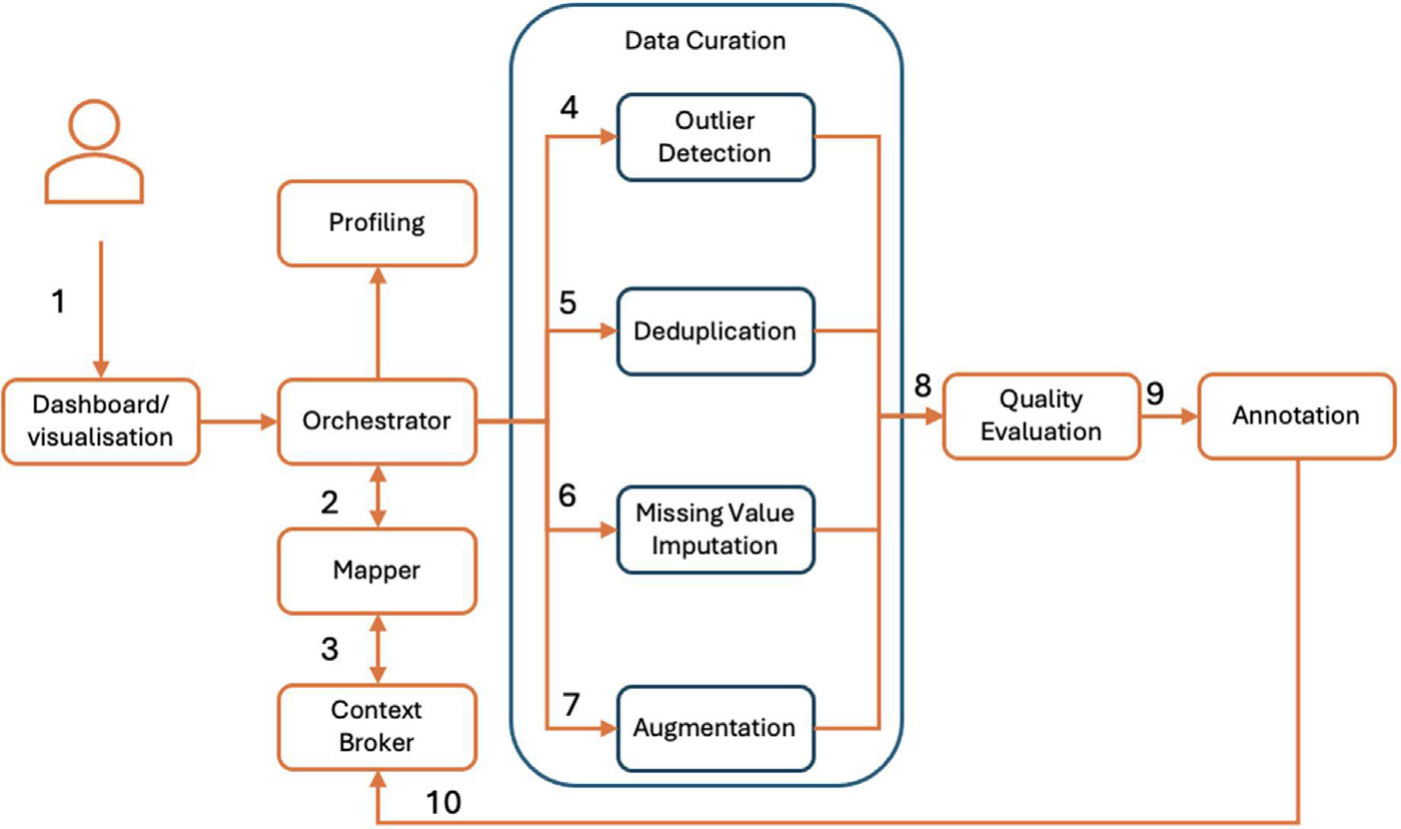
Architecture for data processing pipelines.

As shown in Fig. 3, the pipeline consists of modules for data profiling, curation, quality evaluation, orchestration, feature engineering, semantic enrichment, etc. The main module that manages the overall pipeline is the *data orchestrator*, which translates the user commands and inputs into separate processing steps, running them either in parallel or sequentially by executing the respective modules. Within the current implementation of the SEDIMARK DPP, the *mage.ai* framework [25] is integrated as an orchestrator, providing a simple user interface to define and run the pipeline components.

An example process for the execution of the pipeline is shown in Fig. 3. The sequence of steps is as follows: (i) the user interacts with the data processing dashboard to define the dataset they want to process and the processing actions they want to execute, (ii) the orchestrator executes the step to load the data from storage in various formats (i.e. xls, csv, json) and send it to the Context Broker [26], where it is converted into NGSI-LD format to ensure interoperability across different data sources. This common data model enables seamless integration, allowing data from diverse domains to be processed uniformly.The data is then converted into the proper format (Pandas Dataframe) to be used in the data pipeline, (iii) the pipeline execution steps are applied based on the defined Directed Acyclic Graph (DAG), (iv) the resulting dataset is annotated appropriately and converted back to NGSI-LD to maintain interoperability and (v) the dataset is sent to the Context Broker and either stored locally or shared within the marketplace

Hence, interoperability at data level is supported by employing the NGSI-LD standard for technical interoperability and Smart Data Models for semantic interoperability. Note that the NGSI-LD is currently the only data format supported by SEDIMARK; however, as future work, we aim to extend SEDIMARK to provide an option to data providers to publish data formatters for alternative data formats. Moreover, as it will be presented in Section 4.5, at metadata level, interoperability is supported through the adoption of the SEDIMARK Marketplace ontology, which has been defined leveraging well-known ontologies (e.g. DCAT-AP, ODRL, DCT, etc.). This ontology is used by providers to describe the data and service assets that they make available at the Marketplace.

The Processing steps of the DAG that are executed are for (i) data profiling, identifying statistics about the dataset, the types of columns, ranges of values, etc, (ii) outlier detection, removing outliers, anomalies or noise, applying libraries, such as PyOD [20] and TODS [21] (iii) deduplication, removing duplicate values, applying either method from the RecordLinkage library [27] or using LLM-based techniques [28], (iv) missing value imputation, completing values or rows of values that are missing to convert it to a complete dataset, applying models from Scikit-Learn [29] and (v) data augmentation, augmenting the data with synthetic values for balancing the dataset, removing biases and increasing the fairness of the labels. The final step is data quality evaluation, in which several data quality metrics [30] are used to assess the quality of the data and provide statistics and in-depth insights about the dataset to the user.

Once the data have been cleaned and prepared, they are annotated with meta information extracted during the pipeline process. During the mapping process, the data mapper enriches the data with annotations derived from the previous steps (mainly, the data cleaning one). These annotations are incorporated into the data representation based on the *Data Quality Assessment* model from Smart Data Models^5^, ensuring that the data are reliable and properly annotated for future use following established standards.

### 5.3 AI pipeline and distributed AI for data analytics

**Fig. 4:**
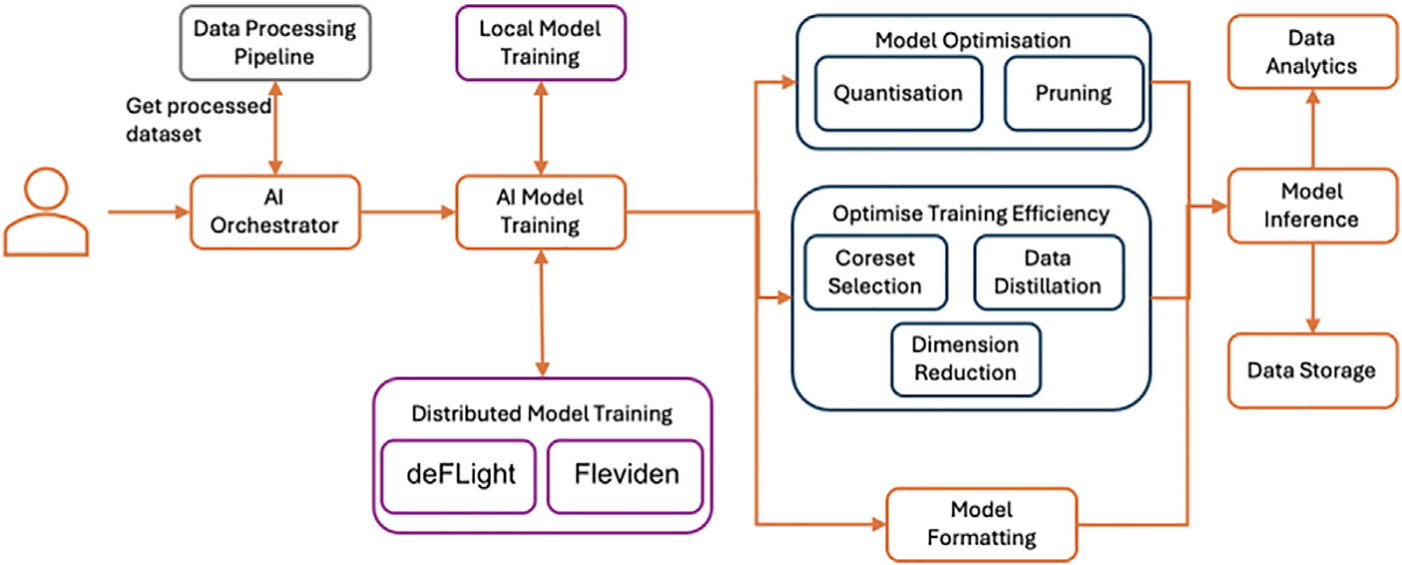
Architecture for AI pipelines.

The AI pipeline in SEDIMARK provides a comprehensive and modular solution for building, training, and optimizing machine learning (ML) models. This pipeline supports both local and distributed training, enabling participants to perform collaborative analytics without compromising data sovereignty. By integrating state-of-the-art methodologies, the AI pipeline ensures efficiency, scalability, and usability for a diverse range of users, from novices to ML experts. Training can be performed both locally, or in a distributed manner with the collaboration of multiple participants. The pipeline, as shown in Fig. 4 handles the complete ML lifecycle, from loading and processing data (in collaboration with the Data Processing Pipeline) to training and optimising ML models. The pipeline is integrated with MLflow [31], a modular framework to track experiments, store metadata, and optimise training resources. Users can monitor key metrics such as accuracy, loss, and resource utilisation.

The AI pipeline of SEDIMARK includes components for model training and for optimising data efficiency during training, employing techniques for (i) coreset selection [32] to identify small representative samples in the data and avoid training models with huge datasets, (ii) data distillation [33] to reduce the dataset sizes by creating smaller synthetic datasets with the same information as the original ones, and (iii) dimension reduction [34] to minimise the dimensions of the feature space. Additionally, the AI pipeline includes techniques for optimising ML models for real-time inference on edge devices with (i) quantisation [35], for decreasing the size of the models and saving energy during inference, and (ii) pruning [36], for sparsifying the computational graph and reducing both inference cost and memory usage.

As discussed in Section 3, decentralisation is a key feature of SEDIMARK. To support this, SEDIMARK employs techniques for the distributed training of machine learning models, so that participants can train models collaboratively using similar datasets, without the need to transfer raw data to a central location. This can be achieved using techniques such as (i) Federated Learning (FL) [37,38] and (ii) Gossip Learning (GL) [[39], [40], [41]].

(i) FL supports decentralised machine learning with the use of a central server that acts as an orchestrator for the training process. At the same time, SEDIMARK users perform local training on their local data (or data purchased from the marketplace) and send model parameters to the central server for aggregation and computation of the global model.

On the other hand, (ii) GL uses a peer-to-peer architecture (without the central server) to carry out the decentralised learning process. In this approach, SEDIMARK users train their local models on their data and only communicate with a subset of local “neighbours”, sending their latest model parameters and then aggregating the parameters they receive from their neighbours.

In SEDIMARK, decentralised AI is supported by two independent frameworks: (i) deFLight and (ii) *FLEVIDEN*. Both frameworks aim to support the modular development of distributed AI approaches in an agnostic way, offering the flexibility to easily choose between the two [42]:

***deFLight***: is a lightweight distributed ML framework supporting both FL and GL. This framework is built in Python and facilitates the distributed AI training process with a straightforward communication protocol for transmitting training instructions, model structures and weights, as well as relevant model metadata.

***FLEVIDEN***: is a flexible and extensible tool for defining computational graphs that represent FL agents and their operations. The core element of FLEVIDEN is the Pod, which connects through input and output wires to form a federated computational graph. The essential operations in FLEVIDEN include creating and linking pods, receiving messages, and sending messages to external systems.

### 5.4 Secure and privacy-preserving data storage and sharing

The SEDIMARK platform handles several storable artefacts that are generated or used by Participants. These artefacts include (i) *data* assets (datasets, data streams, intermediate processing artefacts), (ii) *AI* assets (AI Models, model weights, training descriptions, services), (iii) marketplace assets (participants' Self-Listings - lists of assets they share through the marketplace-, offerings and their cryptographic hashes from Self-Listings, offering descriptions), and (iv) DLT assets (verifying credentials - VCs and smart contracts).

The persistence mechanism adopted for the various artefacts differs. Data Assets are normally stored through either data brokers that adopt an underlying relational store compatible with time-series data, which mainly holds bulk data, or an in-memory means for buffering stream data points. AI Model Assets are stored in an Object store and exposed within the toolbox via an AWS S3-compatible API [43]. Offerings are stored in a document store in the case of Self-listings, and in triple stores in the case of the Catalogue, which are exposed through a SPARQL endpoint [44] and HTTP Graph Protocol [45] interface. For DLT and Trust artefacts, a distributed flat-file system approach is specified, which supports frequent access, such as IPFS [46].

**Fig. 5:**
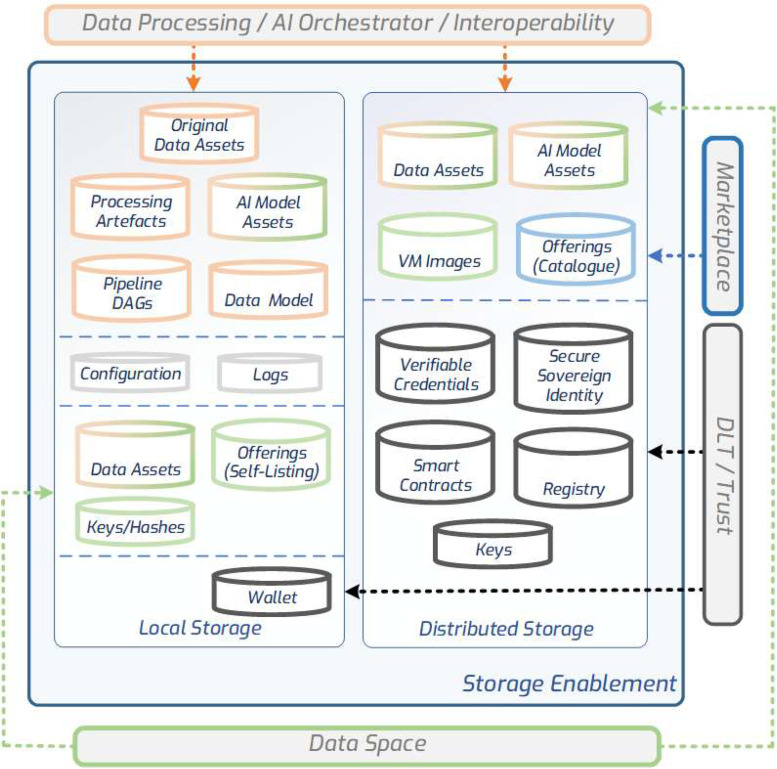
Storage enablement for local and distributed artefacts.

The SEDIMARK platform approaches the storage of Assets and artefacts in a decentralised manner, where, within each node, both local and distributed storage branches exist. As illustrated in Fig. 5, local storage handles the basic storage needs for the data provider to persist their data and AI Model assets, Self-listings for Offering descriptions, and artefacts created throughout the data processing pipeline. The distributed storage element handles the storage of artefacts generated by data providers and is shared through the Marketplace, as well as the Offerings for the Local Catalogue hosted within a Participant's domain, which will be used by the Global Catalogue as a source of Offerings to be advertised. Access to the Assets from outside of the Participant's Toolbox is controlled by the permissions set in the Offering Contracts via the Data Space Enabler (DSE) described below.

### 5.5 Secure and fair access to data

SEDIMARK aims to ensure that exchanged assets are shared securely, with access and usage governed by a transparent and fair set of guidelines that all participants must adhere to. To do so, SEDIMARK defines the Data Space Enabler (DSE), a core component based on the Connector concept from the IDS standard [47], which acts as the primary gateway for any participant interacting with a Marketplace, ensuring secure and trustworthy exchanges of digital assets.

In SEDIMARK, assets are encapsulated within Offerings, which are structured using a JSON-LD [48] semantic data model. Offerings represent assets in a marketplace and provide detailed descriptions of how to access, retrieve, and use them, along with the conditions governing their use. Marketplace participants can then adopt the role of Consumers or Providers, managing interactions involving offerings throughout their entire lifecycle, which can be divided into three main phases:

**Offering Management**: This phase involves activities related to creating and registering Offerings (by Providers) and discovering existing Offerings (by Consumers). While creating an Offering involves semantically formatting an asset description, registration ensures trustworthiness by persisting an endpoint to the Offering and its hash into the SEDIMARK DLT. Interoperability at metadata level is also promoted at this stage through the adoption of the SEDIMARK Marketplace ontology.^6^ It covers the fundamental concepts needed for the registration and the discovery and exchange of offerings and assets. Providers use this ontology to describe their Offerings, which is fundamentally a combination of an asset description and the policies to access that asset, while Consumers can leverage the richness of the underlying model to discover the Offerings that best fit their needs within the catalogue. Extensibility of the model is also ready to host domain-specific attributes which could be used for domain-specific marketplaces with specialized participants such domain-specific search parameters.

**Negotiation**: Before any asset exchange takes place, participants must negotiate the terms and conditions under which the assets in the Offering will be accessed and used. Each Offering includes a set of policies defined through ODRL [49], a data model for digital assets. Consumers may propose modifications to these terms, which Providers can accept or reject. Upon finalising the negotiation, a legally binding agreement is recorded on the DLT, and the relevant authorisation rules are established for the Consumer's access.

**Asset Exchange**: As the final step, Consumers gain access to the digital assets negotiated through DSE, which facilitates the exchange. To this end, the DSE enforces compliance with the agreed-upon rules, ensuring that all negotiated terms are met. SEDIMARK doesn’t deal with Digital Rights Management (DRM) and only protects data access using access control policies enforced at the DSE. SEDIMARK doesn’t deal with Digital Rights Management (DRM) and only protects data access using access control policies enforced at the DSE. Policies ruling the Remote Access control enforced at the DSE are published within the registered Offering (typically described in ODRL) and can be negotiated between the participants, but once the agreement is made, access to the corresponding asset has to conform with such policies.

Fig. 2 illustrates the negotiation and asset exchange architecture. It details the sequential steps between components starting from the initial negotiation phase, where parties agree on the terms and conditions, to the final asset exchange phase, ensuring transparency and security.

### 5.6 Findable data in SEDIMARK

To ensure the compliance with FAIR principles set up by the EU commission, SEDIMARK also incorporates content-based Recommender Systems (RS) [50]. The goal of the RS is to allow users to quickly find relevant datasets within the SEDIMARK platform. This is achieved through two scenarios, (i) using user-defined queries to search for relevant assets; and (ii) finding similar assets based on a specific given asset. Content-based RS produce their recommendations based on certain content associated with an item. In SEDIMARK, each asset is described using an asset metadata and the RS uses this metadata to navigate among available assets to retrieve the most relevant asset based on the user’s need. Therefore, in SEDIMARK, language models are used to perform the content-based RS. In particular, SEDIMARK comes with several RS algorithms based on latent semantic indexing [51] or sentence transformers models [52]. This allows users to flexibly choose between RS based on their needs. Overtime, as SEDIMARK will become more mature and a decent amount of past user interactions will be collected, a collaborative filtering RS models [53] will be added to SEDIMARK. These models take into account past user history and find similar users in terms of behaviour. This in turn will allow the model to recommend more personalised assets without the need of explicit search by the user. Further, similarly as in the decentralised ML training supported by SEDIMARK, the RS will also operate in a decentralised manner, scanning periodically for new assets and storing appropriate indexing of assets in a decentralised way.

### 5.7 Ethical data incentivisation and monetisation schemes

Ethical Data Incentivisation and Monetisation Schemes [54] refer to approaches that encourage data sharing and generate value from data while adhering to ethical principles and respecting individual privacy rights. In the context of European data spaces, these schemes aim to create a framework for responsible data usage that balances economic benefits with ethical considerations.

Ethical data schemes prioritise transparency in data collection and usage practices. Organisations must obtain explicit consent from individuals before collecting and monetising their personal data. This is achieved by clearly explaining how data will be used, with easily understandable privacy notices and offering options for data privacy preferences, including opt-out mechanisms.

To implement these schemes in a marketplace such as SEDIMARK, several aspects must be taken into account. Compliance with the General Data Protection Regulation (GDPR) is fundamental, including obtaining explicit consent for data processing, respecting data subject rights, and maintaining detailed records of data processing activities. For that purpose, data anonymisation and aggregation techniques, such as data masking, encryption, and pseudonymisation, are being proposed as part of the SEDIMARK toolbox to remove personally identifiable information from datasets.

Fair compensation models are made possible to ensure that individuals and enterprises, especially SMEs, are appropriately rewarded for the value their data provides. This could involve monetary compensation, discounts, other incentives, or enhanced services and features, and is identified as a requirement for the SEDIMARK Marketplace enabler.

Finally, regular audits and updates are necessary to ensure ongoing compliance with ethical standards and evolving regulations. This includes reviewing data collection and usage practices, updating privacy policies and consent mechanisms, and adapting to new technologies and consumer needs. The use of a distributed, DLT-based architecture in SEDIMARK contributes to this policy by providing audit trail records of actions made upon datasets.

SEDIMARK also aims to foster transactions between its participants by introducing a rating system, allowing users to provide feedback and reviews on the offerings they consume. Providers are therefore incentivised to improve the quality of their data assets using the data quality pipeline in the SEDIMARK toolbox.

## 6 Conclusion

This paper describes an overview of the architecture of SEDIMARK, which is a decentralised marketplace for sharing data and service assets in data spaces. SEDIMARK has a strong focus on decentralisation, trust, intelligence, interoperability and data quality. SEDIMARK leverages distributed ledger technology (DLT) to ensure the integrity of information by providing trustworthy, non-repudiable, and immutable records of transactions. The architecture consists of components for (i) AI-based data processing and quality improvement, (ii) training and optimising AI models both locally and in a distributed way, (iii) secure, privacy-preserving data storage and sharing, (iv) secure and fair access to data, through the SEDIMARK connector, inspired by the IDSA, (v) improved user experience through a recommender system that enhances asset discovery, and (vi) ethical data incentivisation and monetisation schemes, focusing on creating specific strategies to ensure fair data sharing and value generation, while adhering to rigorous ethical guidelines to promote responsible data usage. The SEDIMARK platform is modular and extensible and caters to different types of users. The SEDIMARK tools are under development and will be released as open-source on the project's GitHub page.^7^

## Ethics Statement

The authors have read and followed the ethical requirements for publication in Data in Brief and confirmed that the current work does not involve human subjects, animal experiments, or any data collected from social media platforms.

## CRediT Author Statement

Erika Duriakova*: methodology, software, writing - review and editing, writing - original draft

Diarmuid O’Reilly-Morgan: methodology, software, validation, writing - review and editing, writing - original draft

Maroua Bahri: writing - original draft

Maxime Costalonga: writing - original draft

Gabriel Danciu: writing - original draft

Septimiu Nechifor: supervision, validation

Tarek Elsaleh: methodology, validation, supervision, funding acquisition, writing - original draft

Peipei Wu: formal analysis, software, validation, writing - original draft

Franck Le Gall: funding acquisition, conceptualization, supervision, writing - original draft

Grigorios Koutantos: writing - original draft

Panagiotis Vlacheas: funding acquisition, conceptualization, methodology, supervision, writing - original draft

Luis Sánchez: funding acquisition, conceptualization, methodology, supervision, writing - original draft

Juan Ramón Santana: methodology, investigation, software, writing - original draft

Pablo Sotres: methodology, investigation, software, writing - original draft

Elias Tragos: funding acquisition, conceptualization, methodology, supervision, writing - review and editing, writing - original draft

## Data Availability

GitHubSEDIMARK (Original data).

## Footnotes

1 https://digital-strategy.ec.europa.eu/en/policies/simpl.

2 http://open-source.i3-market.eu/.

3 http://perledger.org/.

4 https://www.cockroachlabs.com/product/cloud/.

5 https://github.com/smart-data-models.

6 https://sedimark.github.io/ontology/.

7 https://github.com/SEDIMARK.

## References

[1] European Commission: communication from the commission to the European parliament, the council, the European economic and social committee and the committee of the regions a European strategy for data (2020).

[2] European Commission: regulation (EU) 2023/2854 of the European parliament and of the council of 13 December 2023 on harmonised rules on fair access to and use of data and amending regulation (EU) 2017/2394 and directive (EU) 2020/1828(data act) (text with EEA relevance) (2023).

[3] Curry E., Auer S., Berre A.J., Metzger A., Perez M.S., Zillner S. (2022). Technologies and Applications for Big Data Value.

[4] Data spaces support centre: data spaces blueprint v1.0. https://dssc.eu/page/blueprint, Accessed: 2024-09-24.

[5] EU Data Market Monitoring Tool: The European data market monitoring tool key facts and figures, first policy conclusions, data landscape and quantified stories.d2.9 final study report (2024).

[6] Tragos et. al.: SEDIMARK: SEcure Decentralised Intelligent Data MARKetplace, https://sedimark.eu/.

[7] S. Popov: The tangle (2018), https://assets.ctfassets.net/r1dr6vzfxhev/2t4uxvsIqk0EUau6g2sw0g/45eae33637ca92f85dd9f4a3a218e1ec/iota1_4_3.pdf.

[8] C. Mader, J. Pullmann, S. Tramp, C. Lange: International data spaces information model. https://w3id.org/idsa/core, Accessed: 2024-09-24.

[9] International Data Spaces Association: Dataspace Protocol. https://docs.internationaldataspaces.org/ids-knowledgebase/v/dataspace-protocol/, Accessed: 2024-09-24.

[10] Gaia-X European Association for Data and Cloud: Gaia-X Architecture Document. https://gaia-x.gitlab.io/technical-committee/architecture-working-group/architecture-document/, Accessed: 2024-09-24.

[11] Data spaces support centre: data spaces 101. https://dssc.eu/space/SK/32407574/1+Data+Spaces+101, Accessed: 2024-09-24.

[12] Gabrielli S., Krenn S., Pellegrino D., Pérez Baún J.C., Pérez Berganza P., Ramacher S., Vandevelde W. (2022). Data Spaces: Design, Deployment and Future Directions.

[13] OMEGA-X Consortium: orchestrating an interoperable sovereign federated multi-vector energy data space built on open standards and ready for Gaia-X (2023).

[14] Vlacheas et. al. Use cases definition and initial requirements analysis. D2.1(June 2023).

[15] Tragos et. al: SEDIMARK architecture and interfaces. D2.3 (September 2024).

[16] ETSI G. (2019). Context Information Management (CIM); NGSI-LD API. Context Inf. Manag. (CIM).

[17] Drąsutis, E.: Iota smart contracts (2021), https://files.iota.org/papers/ISC_WP_Nov_10_2021.pdf.

[18] M. Sporny, D. Longley, M. Sabadello, D. Reed, O. Steele, C. Allen: Decentralized identifiers (DIDs) v1.0. W3C recommendation, W3C (Jul 2022), https://www.w3.org/TR/2022/REC-did-core-20220719/.

[19] M. Sporny, D. Longley, D. Chadwick: Verifiable credentials data model v1.1. W3C recommendation, W3C (Mar 2022), https://www.w3.org/TR/2022/REC-vc-data-model-20220303/.

[20] Y. Zhao, Z. Nasrullah, Z. Li: Pyod: a python toolbox for scalable outlier detection (2019), https://arxiv.org/abs/1901.0158.

[21] Lai K.H., Zha D., Wang G., Xu J., Zhao Y., Kumar D., Chen Y., Zumkhawaka P., Wan M., Martinez D. (2021). Proceedings of the AAAI Conference on Artificial Intelligence.

[22] Feurer M., Klein A., Eggensperger K., Springenberg J., Blum M., Hutter F., Cortes C., Lawrence N.D., Lee D.D., Sugiyama M., Garnett R. (2015). Advances in Neural Information Processing Systems.

[23] Olson R.S., Bartley N., Urbanowicz R.J., Moore J.H. (2016). Proceedings of the Genetic and Evolutionary Computation Conference 2016. p. 485–492. GECCO ’16, Association for Computing Machinery.

[24] Bahri M., Salutari F., Putina A., Sozio M. (2022). Automl: state of the art with a focus on anomaly detection, challenges, and research directions. Int. J. Data Sci. Anal..

[25] mage.ai Documentation. https://www.mage.ai, accessed: 2024-09-19.

[26] ETSI: Context Information Management (CIM); NGSI-LD API. ETSI GS CIM 009V1.8.1. ETSI (2024).

[27] Sariyar M., Borg A. (2010). The record linkage package: detecting errors in data. R. J..

[28] O’Reilly Morgan D., Tragos E., Duriakova E., Du H., Hurley N., Lawlor A. (2024). New Trends in Database and Information Systems: ADBIS 2024 Short Papers, Workshops, Doctoral Consortium and Tutorials. ACM.

[29] Pedregosa F., Varoquaux G., Gramfort A., Michel V., Thirion B., Grisel O., Blondel M., Prettenhofer P., Weiss R., Dubourg V. (2011). Scikit-learn: machine learning in python. J. Mach. Learn. Res..

[30] Tragos et. al: Energy efficient AI-based toolset for improving data quality. D3.1(December 2023).

[31] Zaharia M., Chen A., Davidson A., Ghodsi A., Hong S.A., Konwinski A., Murching S., Nykodym T., Ogilvie P., Parkhe M. (2018). Accelerating the machine learning lifecycle with mlflow. IEEE Data Eng. Bull..

[32] Feldman Dan. (2020). Core-sets: updated survey. Sampling Techniques for Supervised or Unsupervised Tasks.

[33] Yu Ruonan, Liu Songhua, Wang Xinchao (2023). IEEE Transactions on Pattern Analysis and Machine Intelligence.

[34] Ray Papia, Reddy S.Surender, Banerjee Tuhina (2021). Various dimension reduction techniques for high dimensional data analysis: a review. Artif. Intell. Rev..

[35] Polino, Antonio, R. Pascanu, and D. Alistarh. “Model compression via distillation and quantization.” *arXiv preprint arXiv:1802.05668* (2018).

[36] He Yang, Xiao Lingao (2023). IEEE Transactions on Pattern Analysis and Machine Intelligence.

[37] J. Konečn `y, H.B. McMahan, F.X. Yu, P. Richtárik, A.T. Suresh, D. Bacon,: Federated learning: strategies for improving communication efficiency. arXiv preprint arXiv:1610.05492 (2016).

[38] McMahan B., Moore E., Ramage D., Hampson S., Arcas B.A. (2017). Artificial Intelligence and Statistics.

[39] Kempe D., Dobra A., Gehrke J. (2003). 44th Annual IEEE Symposium on Foundations of Computer Science, 2003. Proceedings.

[40] Tang H., Lian X., Yan M., Zhang C., Liu J. (2018). International Conference on Machine Learning.

[41] Duriakova Erika (2020). Machine Learning and Knowledge Discovery in Databases: European Conference, ECML PKDD 2020.

[42] Tragos et. al.: Enabling tools for data interoperability, distributed data storage and training distributed AI models. D3.3 (January 2024).

[43] Amazon Web Services: S3 API reference. https://docs.aws.amazon.com/amazons3/latest/api/ (Accessed Sept 2024) (2024).

[44] W3C: SPARQL 1.1 protocol. https://www.w3.org/tr/sparql11-protocol/ (Accessed Sept 2024) (2024).

[45] W3C: SPARQL 1.1 graph store HTTP protocol. https://www.w3.org/tr/sparql11-http-rdf-update/ (Accessed Sept 2024) (2024).

[46] IPFS: IPFS standards. https://specs.ipfs.tech/ (Accessed Sept 2024) (2024).

[47] I. Otto, S. Steinbuß, A. Teuscher, I. Lohmann, A. Auer, S. Bader, H. Bastiaansen, H. Bauer, I. Birnstil, M. Böhmer: International data spaces association—reference architecture model—version 3.0 (2019).

[48] M. Sporny, D. Longley, G. Kellogg, M. Lanthaler, & N. Lindström (2020). JSON-LD 1.1. W3C recommendation, Jul.

[49] W3C: ODRl information model 2.2. https://www.w3.org/tr/odrl-model/ (Accessed Sept 2024) (2024).

[50] Javed Umair (2021). A review of content-based and context-based recommendation systems. Int. J. Emerg. Technol. Learn. (iJET).

[51] Rosario Barbara. (2000). Latent semantic indexing: an overview. Techn. Rep. INFOSYS.

[52] Gillioz Anthony (2020). 2020 15th Conference on Computer Science and Information Systems (FedCSIS).

[53] Papadakis Harris (2022). Collaborative filtering recommender systems taxonomy. Knowl. Inf. Syst..

[54] Gade Kishore Reddy (2020). Data analytics: data privacy, data ethics, data monetization. MZ Comput. J..

